# Assessment of GF3 Full-Polarimetric SAR Data for Dryland Crop Classification with Different Polarimetric Decomposition Methods

**DOI:** 10.3390/s22166087

**Published:** 2022-08-15

**Authors:** Meng Wang, Changan Liu, Dongrui Han, Fei Wang, Xuehui Hou, Shouzhen Liang, Xueyan Sui

**Affiliations:** 1 Shandong Academy of Agricultural Sciences, Jinan 250100, China; 2 State Geospatial Information Center, Beijing 100070, China

**Keywords:** Gaofen-3 (GF3) SAR, dryland crops, classification, agriculture, polarimetric decomposition

## Abstract

Crop classification is one of the most important agricultural applications of remote sensing. Many studies have investigated crop classification using SAR data, while few studies have focused on the classification of dryland crops by the new Gaofen-3 (GF3) SAR data. In this paper, taking Hengshui city as the study area, the performance of the Freeman–Durden, Sato4, Singh4 and multi-component decomposition methods for dryland crop type classification applications are evaluated, and the potential of full-polarimetric GF3 data in dryland crop type classification are also investigated. The results show that the multi-component decomposition method produces the most accurate overall classifications (88.37%). Compared with the typical polarization decomposition techniques, the accuracy of the classification results using the new decomposition method is improved. In addition, the Freeman method generally yields the third-most accurate results, and the Sato4 (87.40%) and Singh4 (87.34%) methods yield secondary results. The overall classification accuracy of the GF3 data is very positive. These results demonstrate the great promising potential of GF3 SAR data for dryland crop monitoring applications.

## 1. Introduction

Crop location and distribution are critical for crop parameter estimation and crop yield prediction and drought, flood and disease risk analysis. With the development of remote sensing technology and the availability of remote sensing data, satellite remote-sensing technology has been widely used in crop monitoring, with its advantages of being large-scale, dynamic, timely and having a high-accuracy [1].

However, optical remote-sensing data is vulnerable to foggy, rainy and snowy weathers, which jeopardizes the access of high-quality and continuous data. Synthetic aperture radar (SAR) remote sensing, by contrast, can work all day and adapt to various weathers. SAR has the ability to penetrate vegetation. It can not only obtain the surface information of crops, but also reflect the internal structure information of crops [2]. Microwave remote sensing for vegetation information is completely different from optical sensors.

Early studies focused on obtaining the scattering characteristics of ground objects to a single electromagnetic wave and single polarization mode, and many researchers have employed crop classification based on single-band and single-polarization SAR data according to the different backscattering coefficients of crops [3,4,5,6]. With the development of radar technology, many countries have launched a series of radar satellites, meaning that crop classification was no longer limited to the single polarization. Many researchers have also carried out crop classification based on dual-polarization SAR data [7,8,9]. The combination of a SAR image and an optical image could reduce or eliminate ambiguity, incompleteness, uncertainty and a difference of target objects [10]. Many researchers combined the two kinds of data and achieved higher classification accuracy than a single data source [11,12,13]. In recent years, SAR remote sensing technology has been widely used in crop recognition. For example, Phan et al. (2018) [13] analyzed the X-band SAR data as a function of the ground parameters, and their temporal variations were explained to export backscatter indicators that could be used for rice mapping and for the retrieval of sowing information. Dey et al. (2020) [14] employed the Kennaugh matrix to represent Radarsat-2 SAR data, and used the random forest method to classify crops by using the elements of Kennaugh matrix as classification parameters; the overall classification accuracies of 87.75% and 80.41% were achieved for the time-series SAR data over the Indian and Canadian test sites, respectively. Silva-Perez et al. (2021) [15] proposed the multi-temporal polarimetric SAR change detection based on the polarization information, and these represented the physical changes of the ground object; the results showed that this method for monitoring stages of rice crops provided an application of the method for crop mapping from polarimetric SAR data [15]. Overall, SAR has been widely used in crop classification. In addition, compared with single- and double-polarized data, full polarimetric SAR data could extract the whole polarization matrix, geometric structure, dielectric constant and other information of ground-object targets. Many studies have shown that full-polarimetric SAR data has great advantages in crop classification [16,17,18]. Due to the fact That the polarization decomposition method could effectively extract the scattering characteristics of the target, researchers have proposed various methods of target polarization decomposition in recent decades [19,20]. Based on the polarization decomposition methods, researchers have conducted application research in crop recognition [21,22,23,24,25,26].

China’s first high-resolution SAR satellite, the Gaofen-3 (GF3), was launched in August 2016. GF3 has 12 imaging modes, which is the most diversified in the family of synthetic aperture radar imaging satellites. GF3 is able to switch freely between various imaging modes, take wide-range photographs of the Earth and water bodies, and take detailed photographs of specific areas. The incident angle is between 20 and 41, and the antenna direction can be left or right. The spatial resolution of GF3 is between 1 m and 500 m, and the maximum detection range is 650 km. It can provide full polarization information. Nowadays, many polarimetric SAR satellites have been launched, and the efficient usage of these polarimetric SAR data is becoming a significant problem. An et al. (2018) [27] investigated a new land masking approach for the detecting of ships by using GF3 SAR data. Based on the analysis of the characteristics of ships in GF3 SAR data, Pan et al. (2017) [28] proposed an valid ship-detection method to detect ships with the length ranges from about 70 to 300 m. Liu et al. (2018) [29] conducted experiments of change detection using time-series SAR data acquired by Radarsat-2 and GF3 over the city of Wuhan, and achieved a very good finding. Until now, many studies have investigated crop classification using SAR data, and GF3 has been widely used in fields including marine, land-surface mapping and city monitoring. However, few studies have focused on crop classification by the new GF3 SAR data.

In summary, with the development of polarization decomposition technology, polarization decomposition technology has become more and more widely used for remote-sensing image classification. Few research works had previously been carried out to systematically compare the classification performance between different polarization decomposition methods, especially in the remote-sensing classification of dryland crops using GF3 SAR data.

In this paper, we present a study on the performance of different polarimetric decomposition methods in dryland crop classification using the new GF3 SAR data. Our emphasis is on the inter-comparisons among classification results based on different polarimetric decomposition methods (Freeman–Durden, Sato4, Singh4 and a multi-component decomposition). The organizational structure of this paper is as follows: Section 2 introduces the test site, field investigations, polarimetric SAR decomposition principles and the methods. Section 3 and Section 4 present the experimental results of the crop classification and the discussion, respectively. Finally, the conclusions and future research topics are presented in Section 5.

## 2. Materials and Methods

### 2.1. Test Site and Image Data

**Figure 1:**
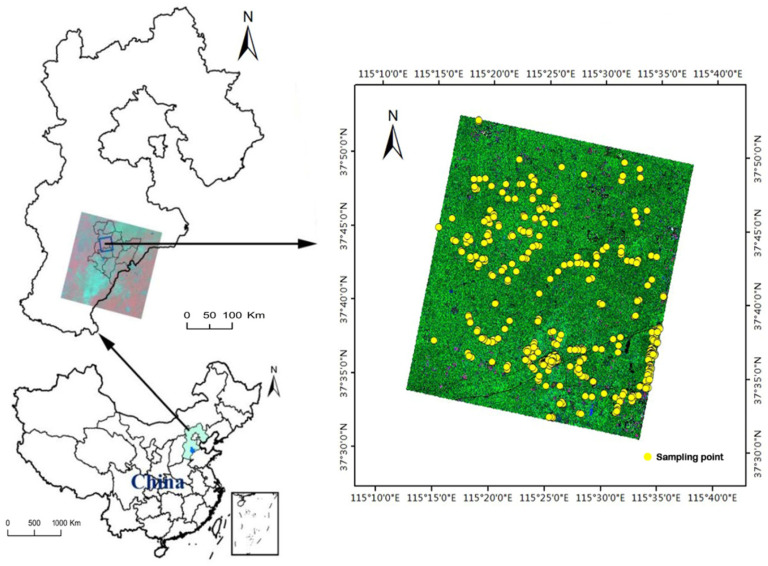
The location of the study area and the distribution of samples.

The study was located in Hengshui (115°10′~116°34′ E, 37°03′~38°23′ N), Hebei province (Figure 1). It was located in the southeastern part of the Hebei province, which belongs to the low plain area and has a total area of 8815 km^2^. The territory is flat and open. It has a semi-humid continental monsoon climate. The average annual temperature is 12 to 13 °C, and the frost-free period lasts for approximately 170 to 220 days. The average annual precipitation is approximately 500–900 mm. The main land types are croplands (with an elevation of approximately 23–29 m), and it has single to double cropping system.

The study area mainly covered 4 types of land cover: corn, cotton, water and a built-up area. The main crop types in the study area are corn and cotton, which comprise approximately 95% of the total agricultural land. The corn planting area was larger than that for cotton, and the distribution was not uniform; the north region of the study area was mainly composed of corn, but small amounts of cotton also existed. Cotton was the main crop in the south region of the study area. Currently, the main crop types are corn and cotton. Corn is planted in May, and harvested in early October. Cotton is planted in June, and harvested in late October. The specific phenological periods of these two types of crops are shown in Table 1 and Table 2.

GF3 images in the full polarized strip mode, imaged on 18 August 2021, were used in this study. The C-band complex data has an incident Angle of 44.635°, a frequency of 5.4 GHz, an azimuth resolution of 5.1975 m, and a range resolution of 2.2484 m. PolSARPro and ENVI were used to process the data sets. The raw data and amplitude format were imported into the coherence matrix. The data were geometrically corrected using the ENVI software. The Refined–Lee speckle filter with a 7 × 7 grid was applied to remove the speckle.

### 2.2. Sampling

We collected ground sample data according to the satellite transition time. In the process of data collection, the methods of systematic sampling and random sampling were employed. Apart from agricultural land, the two other land cover types at the test site are a built-up area and water. The built-up area mainly consists of buildings, roads, and a landscape. The sample plot of different ground scales was obtained by GPS. The size of the cotton sample was about 50 m × 50 m, and the size of the corn sample was about 60 m × 60 m. The actual area of the sample depended on the natural boundary of the field plot. We collected 100 sample sets for each type of land cover (Figure 1), and 70% and 30% of the samples were used for training and validation, respectively.

### 2.3. Principles and Methods

With the development of polarimetric SAR, many polarimetric decomposition methods have been proposed to extract polarimetric information and analyze the scattering mechanisms. Because of its explicit physical meaning and brief form, the decomposition method based on physical scattering model was widely used in recent years, while the most representative decomposition method was the method of Freeman–Durden [30]. This method assumed three typical scattering contributions, and the advantages of this method were its concision and operability. Yamaguchi et al. (2005) [31] proposed a four-component scattering method by adding helix scattering mechanism as the fourth component, and the four-component scattering method was verified by L-Band Pi-SAR data. Since then, Sato et al. (2012) and Singh et al. (2013) proposed three advanced four-component decomposition methods (the Sato4 and Singh4 decomposition methods) for scattering power decomposition [32,33]. Wei et al. (2017) [34] proposed a multi-component decomposition for multi-look polarimetric SAR (PolSAR) data by combining the generalized similarity parameter and the eigenvalue decomposition (the multi-component decomposition method). In addition, there were many other decomposition methods proposed by An (2010), Zyl (2011), Arii (2012), and Wang (2021) et al. [35,36,37,38]. Together, Freeman–Durden, Sato4 and Singh4 were three typical polarization decomposition methods, while the multi-component decomposition method was a representative polarization decomposition method, proposed in recent years. In this paper, the Freeman–Durden, Sato4, Singh4 and multi-component decomposition methods were used to decompose the original image, and the decomposed components were used as the inputs of the supervised classification method. The following sections provided a brief background to these typical polarimetric decompositions.

#### 2.3.1. Freeman–Durden Decomposition Method

The Freeman–Durden decomposition method is a physical scattering model-based decomposition, which describes the polarization backscattering from the naturally existing scatterers. It decomposes the backscattering response into three types: volume scattering (V), the double-bounce scattering (D) and surface or single-bounce scattering, modeled by a first-order Bragg surface scatter (S) [30].

The Freeman–Durden decomposition method can be expressed as follows:

(1) $$\langle \left[T\right]\rangle =f_{s}{\langle \left[T\right]\rangle }_{surface}+f_{d}{\langle \left[T\right]\rangle }_{double}+f_{v}{\langle \left[T\right]\rangle }_{vol}$$

#### 2.3.2. Sato4 Decomposition Method

The Yamaguchi decomposition method extends the Freeman–Durden decomposition method by adding a fourth component: a helix. As reflection symmetry condition cannot be right in some areas of SAR images, the measured coherency matrix is expanded into four submatrices, which correspond to surface, double-bounce, volume and helix scattering mechanisms [31].

(2) $$\langle \left[T\right]\rangle =f_{s}{\langle \left[T\right]\rangle }_{surface}+f_{d}{\langle \left[T\right]\rangle }_{double}+f_{v}{\langle \left[T\right]\rangle }_{vol}+f_{c}{\langle \left[T\right]\rangle }_{helix}$$

Based on the Yamaguchi decomposition method, Sato et al. (2012) proposed a new volume-scattering model that accounts for the HV component caused by double-bounce structures. The new decomposition can be explained in Equation (3) by using the volume-scattering component from vegetation and/or oriented dihedral structures [32]:

(3) $$\begin{array}{l} \langle \left[T^{\prime }\right]\rangle =f_{s}{\langle \left[T\right]\rangle }_{surface}+f_{d}{\langle \left[T\right]\rangle }_{double}+f_{c}{\langle \left[T\right]\rangle }_{helix}\\ +\{\begin{array}{ll} f_{v}{\langle \left[T\right]\rangle }_{vol}^{dipole} & {\;\mathrm{for}\;C}_{1}>0\\ f_{vd}{\langle \left[T\right]\rangle }_{vol}^{dihedral} & {\;\mathrm{for}\;C}_{1}\leq 0 \end{array} \end{array}$$

#### 2.3.3. Singh4 Decomposition Method

Singh et al. (2013) [33] presented a new four-component scattering-power decomposition scheme by implementing a set of unitary transformations for the polarimetric-coherency matrix. The model expansion can be transformed from the rotated basis to the new unitary basis, such that:

(4) $$\begin{array}{l} \langle \left[T^{\prime }\right]\rangle =\langle \left[T(\varphi )\right]\rangle \\ =[U(\varphi )]\left(f_{s}{\langle \left[T\right]\rangle }_{surface}+f_{d}{\langle \left[T\right]\rangle }_{double}+f_{v}{\langle \left[T\right]\rangle }_{vol}+f_{c}{\langle \left[T\right]\rangle }_{helix}\right){\left[U(\varphi )\right]}^{†}\\ =f_{s}{\langle \left[T(\varphi )\right]\rangle }_{surface}+f_{d}{\langle T(\varphi )\rangle }_{double}+f_{v}{\langle T(\varphi )\rangle }_{vol}+f_{c}{\langle T(\varphi )\rangle }_{helix} \end{array}$$

where the † sign denotes the adjoint of the matrix.

#### 2.3.4. Multi-Component Decomposition Method

Wei et al. (2017) proposed a multi-component decomposition method for multi-look polarimetric SAR (PolSAR) data by combining the generalized similarity parameter (GSP) and the eigenvalue decomposition. The proposed method models the rotated coherency matrix [*T*] as a linear sum of five scattering mechanisms (odd-bounce scattering, double-bounce scattering, diffuse scattering, volume scattering and helix scattering) as follows:

(5) $$\begin{array}{l} \langle \left[T\right]\rangle =f_{odd}{\langle \left[T\right]\rangle }_{odd}+f_{dbl}{\langle \left[T\right]\rangle }_{dbl}+f_{diff}{\langle \left[T\right]\rangle }_{diff}\\ +f_{vol}{\langle \left[T\right]\rangle }_{vol}+f_{hlx}{\langle \left[T\right]\rangle }_{hlx} \end{array}$$

The detailed processes of the algorithm are described in [34].

#### 2.3.5. Supervised Classification Method

The basic process of the remote-sensing image supervised classification process includes preprocessing of the original image, selection of the training area, classification of the image, evaluation of the classification accuracy and output of the resulting map. The maximum likelihood ratio classification method, a classical classification algorithm, was used in this study. It is also called the bias classification. The idea is to use the bias classification method as the probability density function to calculate the likelihood of a pixel for each category of the input.

#### 2.3.6. GF3 Polarization SAR Data Processing Method

**Figure 2:**
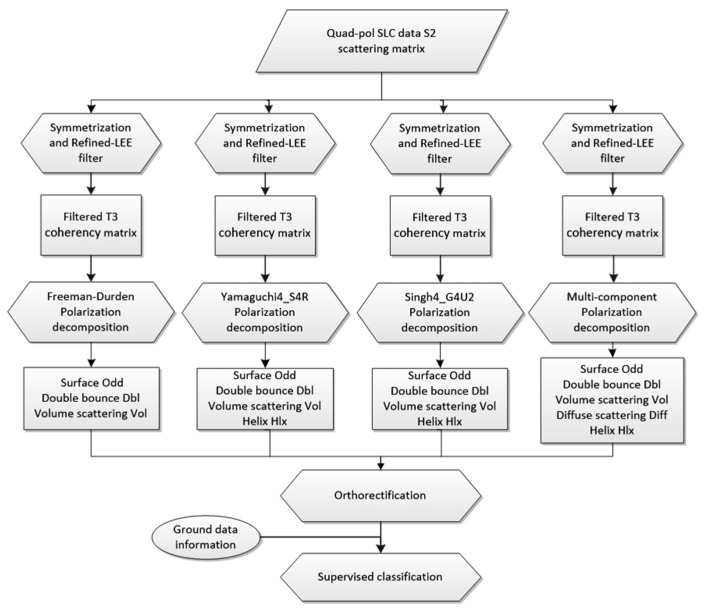
Flowchart of the GF3 polarimetric SAR data processing method.

The GF3 SAR data processing method is as follows: an S2 matrix is generated firstly, PolSARPro is used to process S2 matrix for filtering and then the polarization decomposition is processed. The main flowchart of the GF3 polarimetric SAR data processing is shown in Figure 2.

## 3. Results

### 3.1. RGB Image Composition

**Figure 3:**
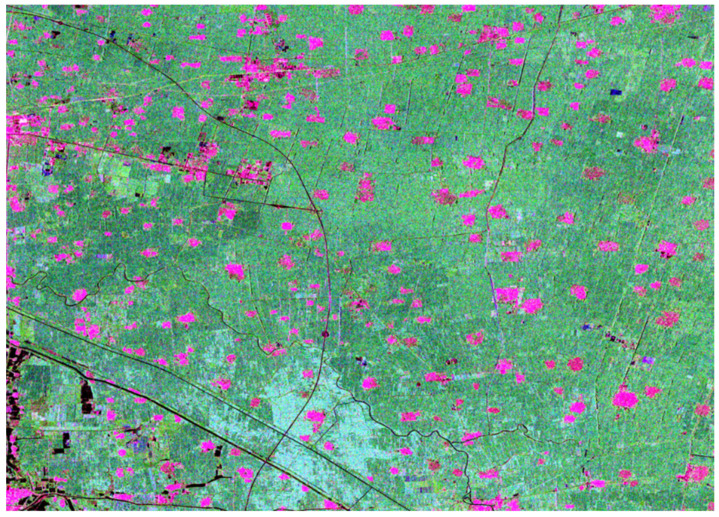
The RGB image composited by multi-component decomposition components (R: Dbl; G: Vol; B: Odd).

First, the fully polarimetric data was filtered to eliminate the coherent noise. Then, we applied the polarimetric decomposition methods on the data to achieve the polarimetric decomposition components. Figure 3 was obtained by the RGB image composited by multi-component decomposition components. It can be seen that it is clear in texture, and the difference between different objects is obvious.

### 3.2. Image Classification

**Figure 4:**
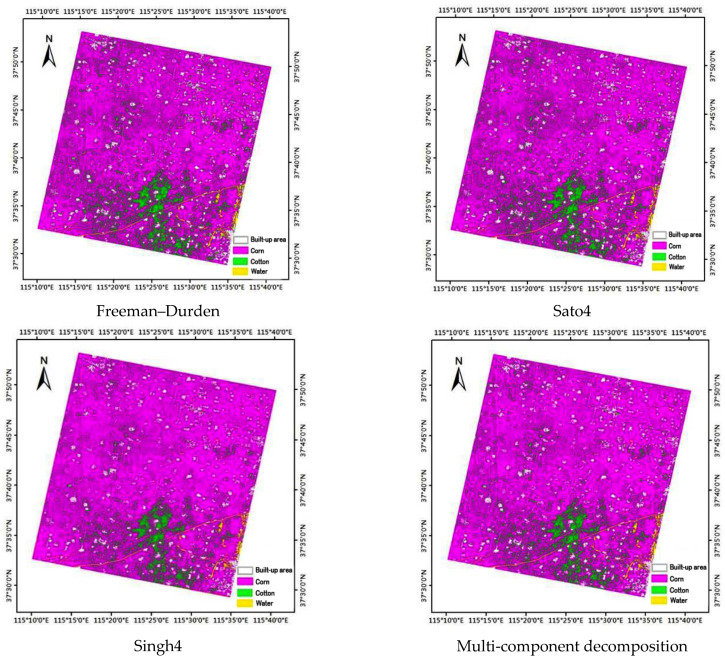
The classification results obtained from the decomposition components using the four polarized decomposition methods (i.e., Freeman–Durden, Sato4, Singh4 and multi-component decomposition).

Using the polarized decomposition components as inputs, we can execute a supervised maximum-likelihood classification. Based on the ground data, regions of interest corresponding to corn, cotton, built-up area, and water were selected as training areas. 100 samples were collected, among which seventy percent of the sample parcels were used as training data and thirty were reserved for validation. Fields were randomly assigned to either training or testing areas, and there was no overlap between the training and testing data. The classification result maps when using the four polarized decomposition methods (i.e., Freeman–Durden, Sato4, Singh4 and multi-component decomposition) are shown in Figure 4.

It can be seen that the white color of the built-up area is enhanced, as compared with other colors in all four results maps. The red color and the green color in the Sato4 and multi-component decomposition results maps have a higher color enhancement, as compared with the Freeman–Durden and Singh4 results maps.

### 3.3. Accuracy Assessment

Accuracy assessments are an extremely important step after information extraction, which is considered a reliable approach for validation. To quantitatively assess the accuracy of crop identification using the methods proposed in this study, the classification accuracy was verified by a confusion matrix method, and the confusion matrix for classification corresponding to each method was calculated. The indexes include the overall accuracy, user accuracy, mapping accuracy and kappa coefficient, which describe the classification accuracy from different perspectives.

The accuracy calculated from the confusion matrix for classification is listed in Table 3. It shows that the accuracy of the overall classification of dryland crop types was higher than 87% using GF3 full polarimetric SAR data.

For the classification results using the Freeman–Durden decomposition method, the mapping accuracies were approximately 94% for corn, 80% for cotton, 78% for urban areas and 98% for water. The kappa coefficient was computed to be approximately 0.81, which was noted to be very good. The mapping results using the Sato4 decomposition method gave a higher overall accuracy of 87.40%. The mapping accuracies were approximately 93.50% for corn, 79.80% for cotton, 79.28% for built-up areas and 98.25% for water, which were relatively higher than those for the Freeman–Durden decomposition method. The kappa coefficient was computed as 0.82 for this classification. The mapping results using the Singh4 decomposition method gave an overall accuracy of 87.34%. The mapping accuracies were approximately 95.95% for corn, 77.36% for cotton, 77.24% for built-up areas and 98.44% for water. The kappa coefficient was computed as 0.82 for this classification, which was relatively similar to the Sato4 decomposition method. The mapping results of the multi-component decomposition method gave the highest overall accuracy of 88.37%. The mapping accuracies were approximately 95.69% for corn, 78.29% for cotton, 79.95% for built-up areas and 98.45% for water. The kappa coefficient was computed as 0.83 for this classification, which was regarded as very high. In Table 3, it can be seen that the misclassification pixels mainly came from confusion between corn and cotton. This misclassification was due to the similar responses to radar signals.

## 4. Discussion

For the recognition accuracy of crop classification, in previous studies, McNairn et al. (2009) [11] used Cloude decomposition, Freeman decomposition and Krogager decomposition to identify corn, soybean and grain near Ottawa by decision-tree classification, wherein the overall classification accuracy reached 88.7%. Jiao et al. (2014) [22] compared the classification accuracy of Cloude decomposition and Freeman–Durden decomposition using the object-oriented classification method, and the overall classification accuracy of wheat, soybean and other crops reached 95%. Qi et al. (2015) [24] proposed a new four-component algorithm, which used the object-oriented method combined with the decision-tree classification method, and its final accuracy was more than 15% higher than the Wishart classification, reaching 86.64%. Huang et al. (2017) [21] used the binary-tree classification method to classify corn, wheat and soybean in western Ontario, Canada, with a final classification accuracy of 87.5% and Kappa coefficient of 0.85. This paper shows that the accuracy of the Freeman–Durden, Sato4, Singh4 and multi-component decomposition methods using full polarimetric SAR data was higher than 87%. Compared with previous studies, the overall accuracy of this paper has reached a higher level. In general, the overall classification accuracy of this paper is very considerable.

In addition, previous studies have mainly focused on rice recognition [2,3,4,5,18], but few on dryland crops which were difficult to identify, and the recognition accuracy of dryland crops was not high. Previous studies mostly used ASAR, ALOS and RADARSAT as SAR data sources [2,3,4,5,11,18,22,24,29], but few studies have been performed on polarization decomposition based on GF3 data. This paper demonstrates the great promising potential of GF3 SAR data for crop-monitoring applications.

Furthermore, due to data availability, the scattering mechanism of crops in different drylands and its change with time series were not investigated in this study, which led to the lack of machine theory and poor universality of the classification algorithm. This should be investigated further.

## 5. Conclusions and Future Research

In this study, we evaluated the dryland crop-type classification capability using different polarimetric decomposition methods. A full polarimetric GF3 polarimetric data set of Hengshui city in China was used to evaluate the Freeman–Durden, Sato4, Singh4 and multi-component decomposition methods for dryland crop type classification application.

Three typical polarization decomposition methods, Freeman–Durden, Sato4 and Singh4, were selected to obtain the surface, dihedral, helix and volume scattering components of dry land use types. In addition, a multi-component decomposition method was used to obtain the surface, dihedral, helix, volume and diffuse scattering components. The parameters derived from the four decomposition approaches were used as the inputs of the maximum-likelihood classification method. The percentage of correctly classified training pixels in the Hengshui city is given for the four polarimetric decomposition methods. Using parameters derived from the polarimetric GF3 data, Sato4, Singh4 and multi-component decomposition method approaches, superior classification accuracies were produced relative to those achieved using the Freeman–Durden decomposition method. Using the multi-component decomposition method, an overall accuracy of above 88% was achieved, which was superior to other three decomposition methods. The Sato4 and Singh4 decomposition methods produced similar, but slightly lower, accuracies. The study has pointed out the following conclusions:

(1) Compared with the typical polarization decomposition method, the accuracy of the classification results using the new decomposition method was highly improved,
(2) The accuracy was effective for dryland crop identification using the GF3 polarimetric data, and it demonstrated the great promising potential of GF3 SAR data for dryland crop monitoring applications.

The decomposition methods selected in this paper were proposed in 2017 at the latest, and further research will be carried out in combination with the latest methods. In order to further extend the use of polarimetric decompositions for monitoring dryland crops, it is necessary to conduct ground-truth verification tests on plant biomasses and canopy water content distribution during SAR data acquisition.

## Figures and Tables

**Table 1:** The specific phenological periods of corn in Hengshui city.

Sowing	Jointing Stage	Heading Stage	Milk-Ripe Stage	Mature Stage
Late May-Middle June	Late June-Middle July	Late July-Early August	Middle August-Middle September	Late September-Early October

**Table 2:** The specific phenological periods of cotton in Hengshui city.

Sowing	Emergence	Squaring	Flowering	Boll-Opening
Middle April-Late April	Early May-Early June	Middle June-Late July	Middle August-Late September	Late September-Early November

**Table 3:** Confusion matrix for the four land-use types using different polarimetric decomposition methods.

Decomposition Methods	Crops or Land Use Type	Mapping Accuracy	User Accuracy	Overall Accuracy	Kappa Coefficient
Freeman–Durden	Corn	94.34%	81.58%	87.20%	0.8139
	Cotton	80.00%	66.31%		
	Built-up areas	77.91%	99.89%		
	Water	98.14%	95.20%		
Sato4	Corn	93.50%	81.53%	87.40%	0.8167
	Cotton	79.80%	66.98 %		
	Built-up areas	79.28%	99.98%		
	Water	98.25%	95.20%		
Singh4	Corn	95.95%	79.86%	87.34%	0.8149
	Cotton	77.36%	73.22%		
	Built-up areas	77.24%	100.00%		
	Water	98.44%	95.21%		
Multi-component	Corn	95.69%	81.16%	88.37%	0.8298
	Cotton	78.29%	75.46%		
	Built-up areas	79.95%	99.99%		
	Water	98.45%	95.21%		

## Data Availability

The data used to support the findings of this study are available from the corresponding author upon request.
